# Localization and function of NO-GC in the murine gastrointestinal and lower urinary tract

**DOI:** 10.1186/2050-6511-14-S1-P37

**Published:** 2013-08-29

**Authors:** Barbara Lies, Dieter Groneberg, Dieter Saur, Andreas Friebe

**Affiliations:** 1 Physiologisches Institut, Universität Würzburg, Germany; 2 Medizinische Klinik (Gastroenterologie), Klinikum rechts der Isar, München, Germany

## Background

Nitric oxide (NO) is well known to stimulate NO-sensitive guanylyl cyclase (NO-GC) followed by production of cGMP and, eventually, to cause smooth muscle relaxation. Recent studies have shown that NO-mediated relaxation of gastrointestinal tissue is dependent on NO-GC in at least two cell types: smooth muscle cells (SMC) and interstitial cells of Cajal (ICC). In addition, the role of NO-GC in a novel interstitial cell type called fibroblast-like cells (FLC) in enteric neurotransmission is debated. In contrast, information about distribution and function of NO-GC in the lower urinary (LU) tract which comprises bladder and urethra is sparse. Here, we examined the function of NO-GC in smooth muscle of the gastrointestinal (GI) and the LU tract.

## Materials and methods

We examined the distribution and function of NO-GC in tissues from the GI and the LU tract of WT and NO-GC-deficient mice (GCKO) as well as in those from animals lacking NO-GC specifically in SMC, ICC or FLC (SM-GCKO, ICC-GCKO, FLC-GCKO). Using immunohistochemistry and isometric force studies we evaluated expression of NO-GC and effects of NO in the different cell types from GI and LU tract. Membrane potential recordings were carried out in fundus and colon. Gut transit time was measured to monitor the consequences of NO-GC deletion on gut motility in vivo.

## Results

In the GI tract, NO-dependent relaxation of GI tissue proceeds via a dual pathway involving SMC and ICC. Tissue from FLC-GCKO mice showed NO-induced relaxation similar to WT. GCKO mice lacked nitrergic relaxation and inhibitory junction potentials. Gut transit time was prolonged in GCKO but, surprisingly, reduced in FLC-GCKO.

In the LU tract, urethral tissue exhibited NO-GC expression exclusively in SMC. Consequently, urethra from WT mice revealed NO-induced relaxation in organ bath experiments which was completely abolished in urethral tissue from GCKO and SM-GCKO mice. Urethra from ICC-GCKO showed a WT-like response. Surprisingly, in bladder smooth muscle, NO-GC expression was detected not in SMC but exclusively in FLC. NO-induced relaxation was absent in bladder strips.

## Conclusion

Our results show that NO-GC is differentially located in the various cells of GI and LU smooth muscle indicating different functional effects of NO/cGMP signaling.

